# Supplementary material for: The Systems Biology Research Tool: evolvable open-source software
Source: BMC Syst Biol. 2008 Jun 29;2:55. doi: 10.1186/1752-0509-2-55 (PMC2446383; doi:10.1186/1752-0509-2-55)
Supplement: Additional file 1 — SBRT Archive. An archive of the current version of the Systems Biology Research Tool. [file 1752-0509-2-55-S1.zip › sbrt-1.4.0/doc/developers_guide/api/sbrt/kernel/math/graph_theory/DirectedGraph.html]

DirectedGraph


|  |  |  |
| --- | --- | --- |
| **Overview** \| **Package** \| **Class** \| **Use** \| **Tree** \| **Deprecated** \| **Index** \| **Help** | |  |
| PREV CLASS   **NEXT CLASS** | **FRAMES**    **NO FRAMES**     **All Classes** |  |
| SUMMARY: NESTED \| FIELD \| CONSTR \| METHOD | DETAIL: FIELD \| CONSTR \| METHOD |  |


---


## sbrt.kernel.math.graph\_theory Class DirectedGraph<N>

```
java.lang.Object
  sbrt.kernel.math.graph_theory.DirectedGraph<N>
```

**Type Parameters:**: `N` - the type of node.

---

``` public class DirectedGraph<N> extends java.lang.Object ```

This class is used to represent directed graphs.

A directed graph is composed of nodes that are connected
by unidirectional edges.

**Author:**
:   This class was written and documented by
    Jeremiah Wright while in the Wagner lab.

---

| **Nested Class Summary** | |
| --- | --- |
| `static class` | `DirectedGraph.Edge<N>`             This class is used to represent the unidirectional edges of a directed graph. |


**Constructor Summary**

`DirectedGraph(java.util.Set<DirectedGraph.Edge<N>> edges)`
  
          Constructs a new directed graph from the provided
collection of edges.


| **Method Summary** | |
| --- | --- |
| `int` | `edges()`             Returns the number of edges in this graph. |
| `java.util.Set<DirectedGraph.Edge<N>>` | `getEdges()`             Returns the set of all edges contained in this graph. |
| `java.util.Collection<N>` | `getNeighbors(java.lang.Object node)`             Returns the neighboring nodes of the provided node. |
| `java.util.Set<N>` | `getNodes()`             Returns the set of all nodes contained in this graph. |
| `java.util.Set<N>` | `getPredecessors(java.lang.Object node)`             Returns the predecessors of the provided node. |
| `java.util.Set<N>` | `getSuccessors(java.lang.Object node)`             Returns the successors of the provided node. |
| `boolean` | `isEdge(java.lang.Object sourceNode, java.lang.Object sinkNode)`             Indicates if an edge exists between the provided nodes. |
| `boolean` | `isNode(java.lang.Object potentialNode)`             Indicates if the provided object is a node in this graph. |
| `int` | `neighbors(java.lang.Object node)`             Returns the number of nodes neighboring the provided node. |
| `int` | `nodes()`             Returns the number of nodes in this graph. |
| `int` | `predecessors(java.lang.Object node)`             Returns the number of predecessors of the provided node. |
| `int` | `successors(java.lang.Object node)`             Returns the number of successors of the provided node. |

**Methods inherited from class java.lang.Object**

`clone, equals, finalize, getClass, hashCode, notify, notifyAll, toString, wait, wait, wait`

**Constructor Detail**

### DirectedGraph

```
public DirectedGraph(java.util.Set<DirectedGraph.Edge<N>> edges)
```

:   Constructs a new directed graph from the provided
    collection of edges.

    **Parameters:**: `edges` - the collection of all edges contained in this graph.


**Method Detail**

### isNode

```
public boolean isNode(java.lang.Object potentialNode)
```

:   Indicates if the provided object is a node in this graph.

    :   **Parameters:**: `potentialNode` - the reference object with which to compare. **Returns:**: `true` if the provided object is a node in this graph; `false` otherwise.

---


### isEdge

```
public boolean isEdge(java.lang.Object sourceNode,
                      java.lang.Object sinkNode)
```

:   Indicates if an edge exists between the provided nodes.

    :   **Parameters:**: `sourceNode` - the source node.: `sinkNode` - the sink node. **Returns:**: `true` if an edge exists between the provided source and sink nodes; `false` otherwise.

---


### getNodes

```
public java.util.Set<N> getNodes()
```

:   Returns the set of all nodes contained in this graph.

    :   **Returns:**: the set of all nodes contained in this graph.

---


### getEdges

```
public java.util.Set<DirectedGraph.Edge<N>> getEdges()
```

:   Returns the set of all edges contained in this graph.

    :   **Returns:**: the set of all edges contained in this graph.

---


### nodes

```
public int nodes()
```

:   Returns the number of nodes in this graph.

    :   **Returns:**: the number of nodes in this graph.

---


### edges

```
public int edges()
```

:   Returns the number of edges in this graph.

    :   **Returns:**: the number of edges in this graph.

---


### neighbors

```
public int neighbors(java.lang.Object node)
```

:   Returns the number of nodes neighboring the provided node.

    :   **Parameters:**: `node` - the node whose number of neighbors will be returned. **Returns:**: the number neighboring nodes of the provided node. **Throws:**: `java.lang.IllegalArgumentException` - if the provided node does not exist in this graph.

---


### getNeighbors

```
public java.util.Collection<N> getNeighbors(java.lang.Object node)
```

:   Returns the neighboring nodes of the provided node.

    :   **Parameters:**: `node` - the node whose neighbors will be returned. **Returns:**: the neighboring nodes of the provided node. **Throws:**: `java.lang.IllegalArgumentException` - if the provided node does not exist in this graph.

---


### successors

```
public int successors(java.lang.Object node)
```

:   Returns the number of successors of the provided node.
    This is also sometimes called the out-degree.

    :   **Parameters:**: `node` - the node whose number of successors will be returned. **Returns:**: the number of successors of the provided node. **Throws:**: `java.lang.IllegalArgumentException` - if the provided node does not exist in this graph.

---


### getSuccessors

```
public java.util.Set<N> getSuccessors(java.lang.Object node)
```

:   Returns the successors of the provided node.

    :   **Parameters:**: `node` - the node whose successors will be returned. **Returns:**: the successors of the provided node. **Throws:**: `java.lang.IllegalArgumentException` - if the provided node does not exist in this graph.

---


### predecessors

```
public int predecessors(java.lang.Object node)
```

:   Returns the number of predecessors of the provided node.
    This is also sometimes called the in-degree.

    :   **Parameters:**: `node` - the node whose number of predecessors will be returned. **Returns:**: the number of predecessors of the provided node. **Throws:**: `java.lang.IllegalArgumentException` - if the provided node does not exist in this graph.

---


### getPredecessors

```
public java.util.Set<N> getPredecessors(java.lang.Object node)
```

:   Returns the predecessors of the provided node.

    :   **Parameters:**: `node` - the node whose predecessors will be returned. **Returns:**: the predecessors of the provided node. **Throws:**: `java.lang.IllegalArgumentException` - if the provided node does not exist in this graph.


---


|  |  |  |
| --- | --- | --- |
| **Overview** \| **Package** \| **Class** \| **Use** \| **Tree** \| **Deprecated** \| **Index** \| **Help** | |  |
| PREV CLASS   **NEXT CLASS** | **FRAMES**    **NO FRAMES**     **All Classes** |  |
| SUMMARY: NESTED \| FIELD \| CONSTR \| METHOD | DETAIL: FIELD \| CONSTR \| METHOD |  |


---
